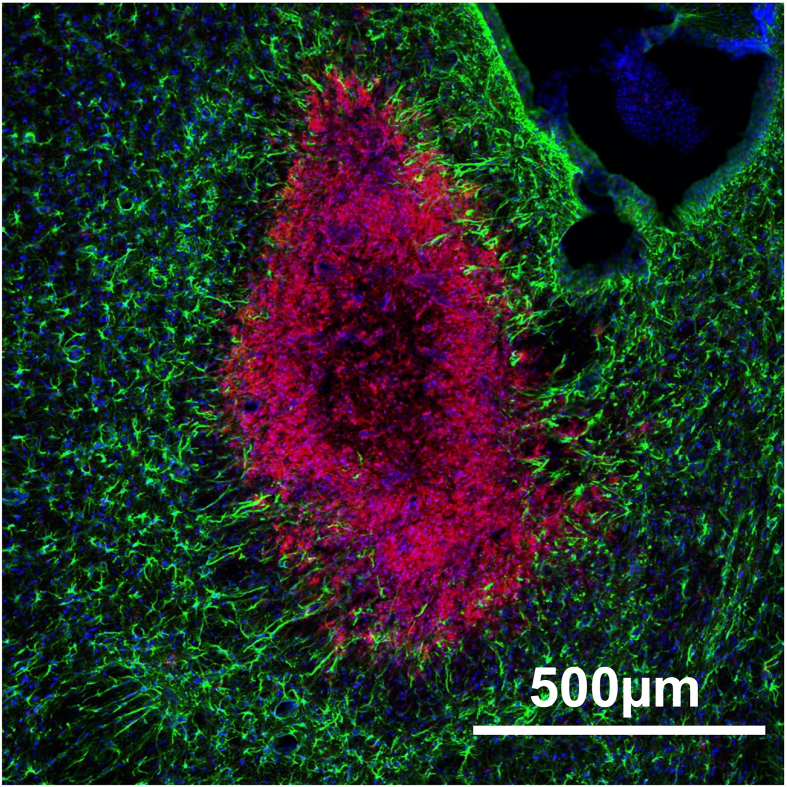# Volatile anaesthetic toxicity in the genetic mitochondrial disease Leigh syndrome

**DOI:** 10.1016/j.bja.2023.08.009

**Published:** 2023-09-26

**Authors:** Kira A. Spencer, Michael Mulholland, John Snell, Miranda Howe, Katerina James, Allison R. Hanaford, Philip G. Morgan, Margaret Sedensky, Simon C. Johnson

**Affiliations:** 1 Center for Integrative Brain Research, Seattle Children's Research Institute, Seattle, WA, USA; 2 Department of Anesthesiology and Pain Medicine, Seattle, WA, USA; 3 Department of Applied Sciences, Translational Bioscience, Northumbria University, Newcastle, UK; 4 Department of Laboratory Medicine and Pathology, Seattle, WA, USA; 5 Department of Neurology, University of Washington, Seattle, WA, USA

**Keywords:** electron transport chain complex I, mitochondrial disease, neurodegenerative disease, paediatric disease, toxicity

## Abstract

**Background:**

Volatile anaesthetics are widely used in human medicine. Although generally safe, hypersensitivity and toxicity can occur in rare cases, such as in certain genetic disorders. Anaesthesia hypersensitivity is well-documented in a subset of mitochondrial diseases, but whether volatile anaesthetics are toxic in this setting has not been explored.

**Methods:**

We exposed *Ndufs4*(−/−) mice, a model of Leigh syndrome, to isoflurane (0.2–0.6%), oxygen 100%, or air. Cardiorespiratory function, weight, blood metabolites, and survival were assessed. We exposed post-symptom onset and pre-symptom onset animals and animals treated with the macrophage depleting drug PLX3397/pexidartinib to define the role of overt neuroinflammation in volatile anaesthetic toxicities.

**Results:**

Isoflurane induced hyperlactataemia, weight loss, and mortality in a concentration- and duration-dependent manner from 0.2% to 0.6% compared with carrier gas (O_2_ 100%) or mock (air) exposures (lifespan after 30-min exposures ∗*P*<0.05 for isoflurane 0.4% *vs* air or *vs* O_2_, ∗∗*P*<0.005 for isoflurane 0.6% *vs* air or O_2_; 60-min exposures ∗∗*P*<0.005 for isoflurane 0.2% *vs* air, ∗*P*<0.05 for isoflurane 0.2% *vs* O_2_). Isoflurane toxicity was significantly reduced in *Ndufs4*(−/−) exposed before CNS disease onset, and the macrophage depleting drug pexidartinib attenuated sequelae of isoflurane toxicity (survival ∗∗∗*P*=0.0008 isoflurane 0.4% *vs* pexidartinib plus isoflurane 0.4%). Finally, the laboratory animal standard of care of 100% O_2_ as a carrier gas contributed significantly to weight loss and reduced survival, but not to metabolic changes, and increased acute mortality.

**Conclusions:**

Isoflurane is toxic in the *Ndufs4*(−/−) model of Leigh syndrome. Toxic effects are dependent on the status of underlying neurologic disease, largely prevented by the CSF1R inhibitor pexidartinib, and influenced by oxygen concentration in the carrier gas.

**Editor’s key points**

• The authors investigated the toxicity of volatile anaesthesia in a mitochondrial disorder.
• Using a mouse model of Leigh Syndrome, volatile anaesthetic exposure was associated with weight loss and mortality.
• Macrophage/microglia depletion reversed volatile anaesthetic-associated toxicity.
• Further research is required to define the critical neuroimmune interactions that determine toxic outcomes from volatile anaesthetic exposure in mitochondrial disorders.

Volatile anaesthetic agents (VAs) are widely used in modern medicine, but the mechanisms underlying their clinically useful effects of sedation, analgesia, paralysis, and amnesia (anaesthetic effects) remain incompletely resolved. In addition, VAs induce many pleiotropic effects, the mechanisms of which are often unknown.^1^

While VAs are remarkably safe in the majority of cases, toxicities impact some populations. Age-related toxicities resulting from VA exposure have been reported in both neonatal and geriatric settings.^2^^,^^3^ VA sensitivity resulting from certain genetic defects is rare but well-documented; significant hypersensitivity to the anaesthetic effects of VAs occurs in patients with mitochondrial disease, particularly those with electron transport chain complex I (ETC CI) defects.^4^, ^5^, ^6^ While VA hypersensitivity is an evolutionarily conserved consequence of ETC CI dysfunction from nematodes to humans, the possibility that VAs might induce acute or chronic toxicities in mitochondrial disease has not been rigorously explored.

Leigh syndrome, a genetically heterogeneous disease, is the most common paediatric presentation of mitochondrial disease.^7^ Defects in NDUFS4, encoding a structural/assembly component of ETC CI, are one cause of *Leigh syndrome*, and the *Ndufs4*-deficient *Ndufs4* knockout (*Ndufs4*(−/−)) mouse provides a remarkably close model for the human disease.^8^ *Ndufs4*(−/−) mice and human patients present with complex multisystem disease involving metabolic dysregulation, neurologic symptoms, and characteristic rapidly progressive symmetric neuroinflammatory lesions in the brainstem.^8^, ^9^, ^10^, ^11^, ^12^, ^13^

*Ndufs4*(−/−) mice are born healthy, as are many patients with mitochondrial disease, with no overt symptoms of neurologic dysfunction before disease onset. Disease typically occurs in the second year of life in humans, while onset in *Ndufs4*(−/−) occurs around postnatal day 37 (P37). Weight loss, abnormal gait, ataxia, and forelimb clasping appear in rapid succession after P37, and median and maximum survival in untreated mice are ∼60–65 and ∼80 days, respectively. Hypersensitivity to anaesthesia by VAs is present at all ages in the *Ndufs4*(−/−); exposure to concentrations appropriate for anaesthesia in normal animals is acutely lethal to *Ndufs4*(−/−) animals.^14^^,^^15^

Management of mitochondrial disease often requires procedures necessitating anaesthesia. Interventions include gastrostomy, cataract surgery, thyroid lobectomy, lipoma excision, cochlear implantation, amongst others.^16^, ^17^, ^18^, ^19^, ^20^, ^21^ Complications after anaesthesia have been reported, but the rarity of the disease, genetic and clinical heterogeneity of patients, *ad hoc* nature of disease management, and comorbid neurologic and metabolic sequelae greatly limit efforts to define VA-induced toxicities in the clinic. Accordingly, animal studies are of great utility for probing disease mechanisms and testing the effects of anaesthetics.

We recently reported that brief VA exposure causes acute hyperlactataemia in the *Ndufs4*(−/−), but the impact of VA exposure on disease progression has not been explored.^8^ Here, we exposed *Ndufs4*(−/−) mice to varying concentrations of isoflurane at pre- and post-disease onset ages to directly assess the impact of anaesthesia with VAs in ETC CI mitochondrial disease. We find exposures to isoflurane cause acute toxic sequelae and accelerate mortality in a concentration- and duration-dependent manner. The effects are strongly influenced by disease status and carrier gas oxygen concentration. Toxic effects are significantly reduced in animals exposed before the onset of CNS disease. Reducing carrier gas oxygen limits chronic, but not acute, toxic sequelae. Finally, treatment with the leukocyte depleting drug PLX3397/pexidartinib prevents both isoflurane and oxygen toxicities in *Ndufs4*(−/−) animals. These data provide new insight into impact of VA exposure in mitochondrial disease and the mechanisms underlying toxic effects.

## Methods

Additional methods are presented in the Supplementary Material.

### Ethics statement and animal use

All experiments were approved by the Institute Animal Care and Use Committee at Seattle Children's Research Institute (Seattle, WA) under protocols IACUC00611 and IACUC00070. The *Ndufs4* knockout mouse line (Jackson Laboratory strain #027058) was obtained from the Palmiter laboratory, University of Washington (Seattle, WA, USA).

All experiments contain approximately the same numbers of male and female mice of each genotype. The *Ndufs4* deletion is recessive, and heterozygosity results in no reported phenotypes and no detectable defects in ETC CI activity. Accordingly, ‘control’ cohorts include both *Ndufs4*(+/−) and *Ndufs4*(+/+) mice.

*Ndufs4*(−/−) animals were housed with control littermates for warmth and stimulation in all studies. Mice were weighed and health assessed a minimum of three times per week. Wetted chow was provided to cages housing *Ndufs4*(−/−) mice displaying neurologic symptoms to ensure food and water accessibility. Humane euthanasia criteria included 20% loss of body weight from maximum or the acute presentation of severe motility or neurologic symptoms perceived to impair access to food or water (immobility, prostrate posture, or otherwise moribund in appearance).

### Animal diets

Breeders and experimental mice were fed PicoLab (Lab Diets, St. Louis, MO, USA) diets 5053 and 5058, respectively. Pexidartinib/PLX3397 chow was prepared as described^8^ (see Supplementary methods).

### Anaesthesia

**Fig 1:**
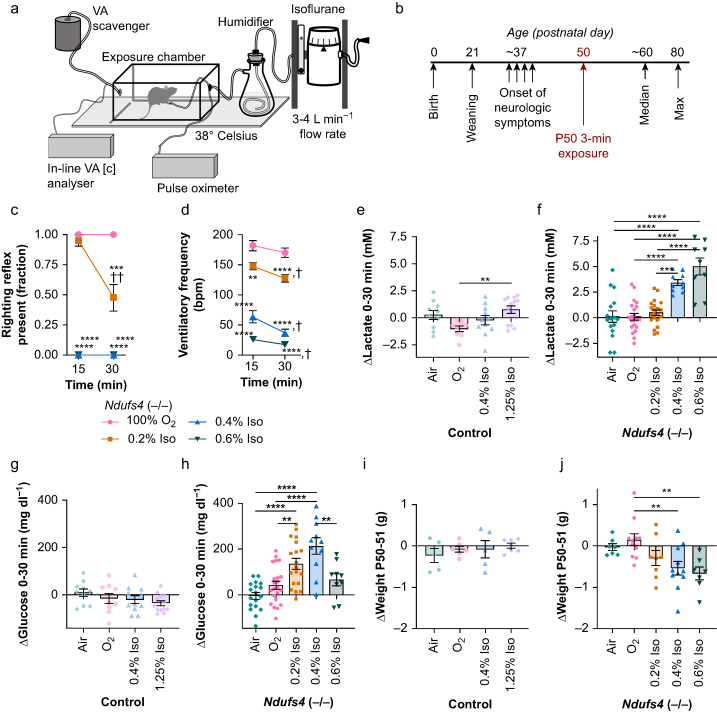
Brief isoflurane exposures cause respiratory depression, metabolic changes, and weight loss in *Ndufs4*(−/−) mice. (a) Schematic of mouse anaesthesia exposure chamber with major components indicated, see also Methods. (b) Overview of the course of disease onset in untreated *Ndufs4*(−/−) animals and the paradigm for testing VA toxicity. Animals are born healthy, beginning to show neurological symptoms around postnatal day 37 (P37). Median and maximum survival in untreated *Ndufs4*(−/−) mice are ∼P60 and ∼P80, respectively. Animals were exposed to isoflurane (Iso), carrier gas (O_2_ 100%), or air in matched conditions, for 30 min at P50, a post-symptom onset age. (c) Righting reflex at 15 and 30 min of exposure. 1=righting reflex is present (animals are unanaesthetised), 0=absent (animals are anaesthetised by this measure). ∗∗*P*<0.01, ∗∗∗*P*<0.0005, ∗∗∗∗*P*<0.00005 compared with oxygen 100% at the matched time, ^††^*P*<0.005 15 *vs* 30 min of exposure, by Mann–Whitney test. (d) Ventilatory frequency at 15 and 30 min of exposure. ∗∗*P*<0.005, ∗∗∗*P*<0.0005, ∗∗∗∗*P*<0.00005 compared with the O_2_ 100% group at the same time by Welch's *t*-test. ^†^*P*<0.05, ^†††^*P*<0.0005 by pairwise *t*-test against 15 min timepoint in the same treatment group. (c–d) *n*≥5 per group, see Supplementary Figure S2 for control animal data. (e) Change in blood lactate concentration in control mice during a 30-min exposure at P50 to Iso 1.25% or 0.4% (equipotent/anaesthetising and equimolar compared with *Ndufs4*(−/−), respectively), O_2_ 100%, or air. One-way analysis of variance (anova) ∗*P*<0.05. (f) Change in blood lactate concentration in *Ndufs4*(−/−) mice during a 30-min exposure at P50 to Iso 0.2%, 0.4%, or 0.6%, O_2_ 100%, or air. One-way anova ∗∗∗∗*P*<0.0001. (g) Change in blood glucose concentration in control mice during a 30-min exposure at P50 to Iso 1.25% or 0.4% (equipotent/anaesthetising and equimolar, respectively), O_2_ 100%, or air. One-way anova=not significant. (h) Change in blood glucose concentration in *Ndufs4*(−/−) mice during a 30-min exposure at P50 to Iso 0.2%, 0.4%, or 0.6%, O_2_ 100%, or air. One-way anova ∗∗∗∗*P*<0.0001. (e–h) *n*≥7 per group. (i) Change in control animal weight over the 24 h after a single 30-min exposure at P50 to Iso, O_2_ 100%, or air. One-way anova=not significant. (j) Change in *Ndufs4*(−/−) weight over the 24 h after a single 30-min exposure at P50 to Iso, O_2_ 100%, or air. One-way anova ∗*P*<0.05. (i–j) *n*≥5 per group. (c–j) Error bars=standard error of the mean (sem) centred on the mean. All datapoints represent biological replicates (individual animals). Any pairwise comparisons not shown are non-significant (*P*>0.05). (e–j) *P*-values shown are Tukey's multiple testing corrected *P*-values: ∗∗∗∗*P*<0.0001, ∗∗∗*P*<0.0005, ∗∗*P*<0.005, ∗*P*<0.05. All datapoints shown. VA, volatile anaesthetic.

Isoflurane (cat. no. 14043070406, Patterson Veterinary, Saint Paul, MN, USA) was provided at concentrations indicated using a routinely calibrated isoflurane vaporiser (Summit Anaesthesia Solutions, Salt Lake City, UT, USA) at a flow rate of 1.5–2 L min^−1^ with an in-line humidifier (Fig. 1). Isoflurane concentration was monitored using an in-line VA analyser AA-8000 (BC Biomedical, Surrey, BC, Canada). O_2_ 100% or medical air were used as carrier gas as specified in individual experiments. The plexiglass exposure chamber and humidifier were pre-warmed to and held at 38°C throughout exposures using a circulating water heating pad HTP-1500 (Adroit Medical, Loudon, TN, USA). Mice were fed *ad libitum* before and after exposures.

Isoflurane was varied from 0% to 0.6% in *Ndufs4*(−/−) experiments. In control mice, two different control concentrations were used where relevant: 1) isoflurane 0.4%, an ‘equimolar’ concentration matching an anaesthetic concentration in *Ndufs4*(−/−)s. This concentration does not anaesthetise control animals. 2) isoflurane 1.25%, an ‘equipotent’ concentration which anaesthetises control mice. Acute effect datasets (P50 and P30) represent the first exposure in the respective repeat (once daily) exposure paradigms.

### Postanaesthesia monitoring

After anaesthesia, mice were placed into a clean cage sitting on a water heating pad and monitored for seizures and emergence from anaesthesia. Observation was concluded when mice were deemed alert by the researcher or 20 min had elapsed, whichever was longer for a given mouse.

### Righting reflex, ventilatory frequency, SpO_2_, and heart rate

Righting reflex, a well-established method for measuring anaesthetic state in mice, was assessed using standard methods.^22^, ^23^, ^24^ Animals were tilted on their side, with righting reflex considered intact if the animal regained a position where all four paws face the ground within 10 s. Righting reflex measures allow for assessment of animal anaesthesia without disruption of gas concentration or flow through the plexiglass anaesthesia chamber. Importantly, loss of righting reflex (LORR) occurs at lower concentrations than loss of response to tail clamp or pedal withdrawal. Accordingly, any toxicities observed at the lowest concentrations causing LORR are relevant to these measures of greater anaesthetic depth.^25^^,^^26^

Ventilatory frequency was assessed by counting breaths during a 15–30 s interval. Peripheral blood oxygen saturation, SpO_2_, and heart rate were monitored by pulse oximetry using Kent Scientific (Torrington, CT, USA) MouseSTAT Pulse Oximeter and Heart Rate modules attached to a PhysioSuite monitor. These monitors utilise paw pad pulse oximeters. Values are impacted by movement and tend to be variable in alert/unanaesthetised animals. Accordingly, high variance in unanaesthetised data and early in anaesthetic exposures (when animals are not yet fully anaesthetised) is expected.

### Blood point-of-care data

Blood metabolites (glucose, β-hydroxybutyrate [βHB], and lactate) were collected using point-of-care meters and the minimally invasive tail-prick method. Values were measured using Prodigy Autocode glucose meters (Prodigy Diabetes Care LLC, Charlotte, NC, USA, product #51850–3466188), Precision Xtra XEGW044 meters with βHB strips (Abbott Laboratories, Chicago, IL, USA), and Nova Biomedical Lactate meters (Nova Biomedical, Waltham, MA, USA, product #40828). We have previously validated the accuracy of these point-of-care meters.^9^

### Experimental design and statistical analyses

Controls were spread chronologically throughout the experiments. Animals were randomly assigned to treatment groups. All exposures were performed at approximately the same time of day to avoid variance that differences in circadian cycle might introduce.

All statistical analyses were performed using GraphPad Prism (GraphPad Software Inc., San Diego, CA, USA) as detailed in figure legends. Details regarding statistical tests, power calculations, and experimental design are provided in figure legends and supplementary materials (Supplementary Methods). There were no differences between male and female animals in any dataset, with the exception of raw weights in control animals (as expected). Where shown in Supplementary data, control mouse raw weights are split into male and female groups. *Ndufs4*(−/−) mice do not show differences between male and females, including weights.^8^, ^9^, ^10^, ^11^, ^12^, ^13^

## Results

### Acute impact of a single exposure to isoflurane in *Ndufs4*(−/−)

To determine whether VA exposure leads to toxic sequelae in the setting of ETC CI mitochondrial disease, we established an experimental paradigm whereby *Ndufs4*(−/−) animals were exposed to isoflurane 0.2%, 0.4%, or 0.6% in oxygen 100% (O_2_ 100%); O_2_ 100% (carrier gas) only; or air (mock exposure) for 30 min once per day for 3 days starting at P50 (Fig. 1a), while control animals were exposed to an equimolar (0.4%) or equipotent (anaesthetising) 1.25% concentration for comparison.

Heart rate and peripheral capillary oxygen saturation (SpO_2_) were maintained during these exposures for all treatment groups (Supplementary Fig. S1). Overall anaesthetic state was judged by righting reflex.^22^, ^23^, ^24^ All *Ndufs4*(−/−) mice exposed to isoflurane 0.4% or 0.6% lost righting reflex by 15 min of exposure at P50, while none exposed to 0.2% lost righting at 15 min and only 50% had by 30 min (Fig. 1c). Ventilatory frequency depression occurred in *Ndufs4*(−/−) mice in a concentration- and time-dependent manner (Fig. 1d).

Metabolic dysregulation is a prominent feature of mitochondrial disease. VAs are known to disrupt circulating metabolite homeostasis in certain conditions.^1^ In *Ndufs4*(−/−) mice exposed to isoflurane, blood lactate is increased by isoflurane in a concentration-dependent manner compared with both O_2_ 100% and air (mock) exposures. Lactate was slightly increased in controls exposed to isoflurane 1.25% compared with O_2_ 100% (which slightly but nonsignificantly reduced lactate compared with air), but not compared with mock (air) exposures (Fig. 1e and f). Significant concentration-dependent increases in blood glucose also occurred in *Ndufs4*(−/−) mice exposed to isoflurane 0.2% and 0.4% *vs* air and O_2_ 100%. Interestingly, glucose was not elevated by isoflurane 0.6%. No treatment significantly altered glucose concentrations in control animals (Fig. 1g and h). βHB was generally unchanged by isoflurane in both control and *Ndufs4*(−/−) mice.

**Fig 2:**
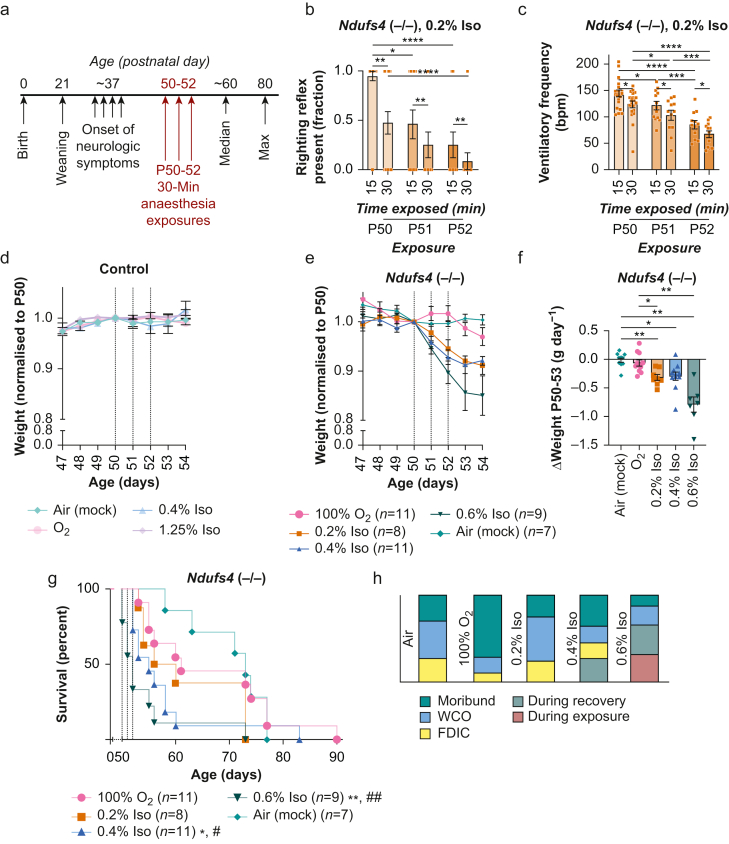
Repeated brief isoflurane exposures increase anaesthesia sensitivity, cause weight loss, and accelerate mortality in a concentration-dependent manner in *Ndufs4*(−/−) mice. (a) Overview of *Ndufs4*(−/−) disease course and the paradigm for assessing the impact of repeated anaesthesia exposures. Animals were exposed to isoflurane, or air or carrier gas (O_2_ 100%) in matched conditions, for 30 min once per day on P50, P51, and P52, with exposures at approximately the same time each day (see Methods). (b) Loss of righting reflex by exposure day and time exposed in isoflurane 0.2%-exposed *Ndufs4*(−/−) animals. ∗∗*P*<0.005 (exposure number factor) and ∗∗∗∗*P*<0.00005 (time factor) by Mann–Whitney test (see Methods). (c) Ventilatory frequency by time into exposure and exposure number in isoflurane 0.2%-exposed *Ndufs4*(−/−) animals. Two-way analysis of variance (anova): time factor ∗∗∗*P*<0.0005, exposure number factor ∗∗∗∗*P*<0.0001. *P*-values shown are Tukey's multiple comparisons test ∗*P*<0.05, ∗∗*P*<0.005, ∗∗∗*P*<0.0005, ∗∗∗∗*P*<0.0001. *P*-values for irrelevant comparisons (different duration and day) not shown. See Supplementary Figure S2 for equivalent comparisons of other treatment groups. (d–e) Plot of control (d) and *Ndufs4*(−/−) (e) weights normalised (by individual animals) to P50, focused on the days spanning the exposures. Plotted are mean with standard error of the mean (sem). Controls—no differences between groups on any day. *Ndufs4*(−/−)—significance of differences between groups not assessed at individual days; rather, the rate of weight change was assessed in (f). (f) The rate of change in weight (grams per day, g day^−1^) in data from (e) during the P50–P53 period, calculated using a slope equation with available datapoints (i.e. where animals did not survive to P53, the slope was calculated using available days). One-way anova: ∗∗∗∗*P*<0.00001. (b–f) Datapoints represent individual animals, with bars at the mean and error bars representing sem. *P*-values shown are Tukey's multiple testing corrected *P*-values, ∗*P*<0.05, ∗∗*P*<0.005. *n*≥6 in each group. (g) Survival of *Ndufs4*(−/−) animals in the P50–P52 30-min exposures. ∗*P*<0.05 *vs* O_2_ 100%, ^#^*P*<0.05 *vs* air (mock) treatment, ∗∗*P*<0.005 *vs* O_2_ 100%, ^##^*P*<0.005 *vs* air (mock) treatment, Gehan–Breslow–Wilcoxon test. (h) Cause of death in *Ndufs4*(−/−) mice from these experiments (those animals in survival curves in [d]). No mortality was observed in any control cohort in these experiments. FDIC, found dead in cage; ISO, isoflurane; WCO, euthanised as a result of reaching weight cut-off.

Weight loss was observed to occur in a concentration-dependent manner after a single exposure in *Ndufs4*(−/−) but not control animals (Fig. 1i and j). Acute mortality occurred in the isoflurane 0.6% cohort (two of nine animals, Fig. 2).

### Toxic effects of repeat anaesthesia exposures in the Ndufs4(−/−) model

**Figure S2:**
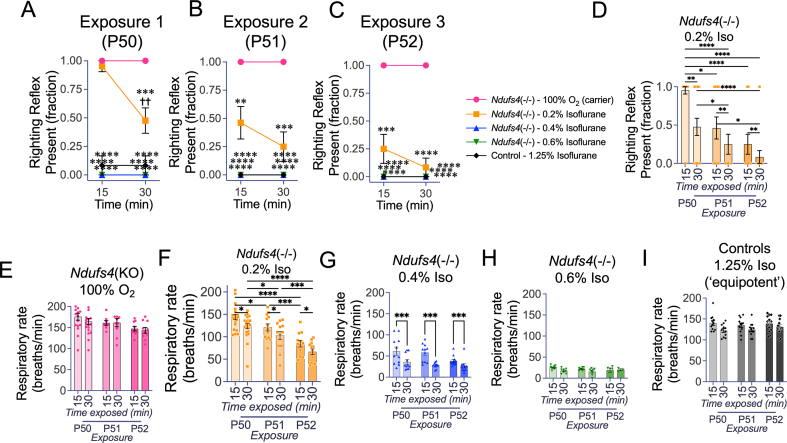


To define the impact of repeat anaesthesia exposures, we exposed these animals again at P51 and 52 (Fig. 2a). Strikingly, isoflurane 0.2% had a significantly greater effect on righting reflex and ventilatory frequency depression on second and third exposures (Fig. 2b and c, Supplementary Fig. S2; higher concentrations had a maximal effect during each exposure). In control mice, isoflurane 1.25% is sufficient to anaesthetise but did not lead to ventilatory frequency depression in any exposure (Supplementary Fig. S2).

**Figure S3:**
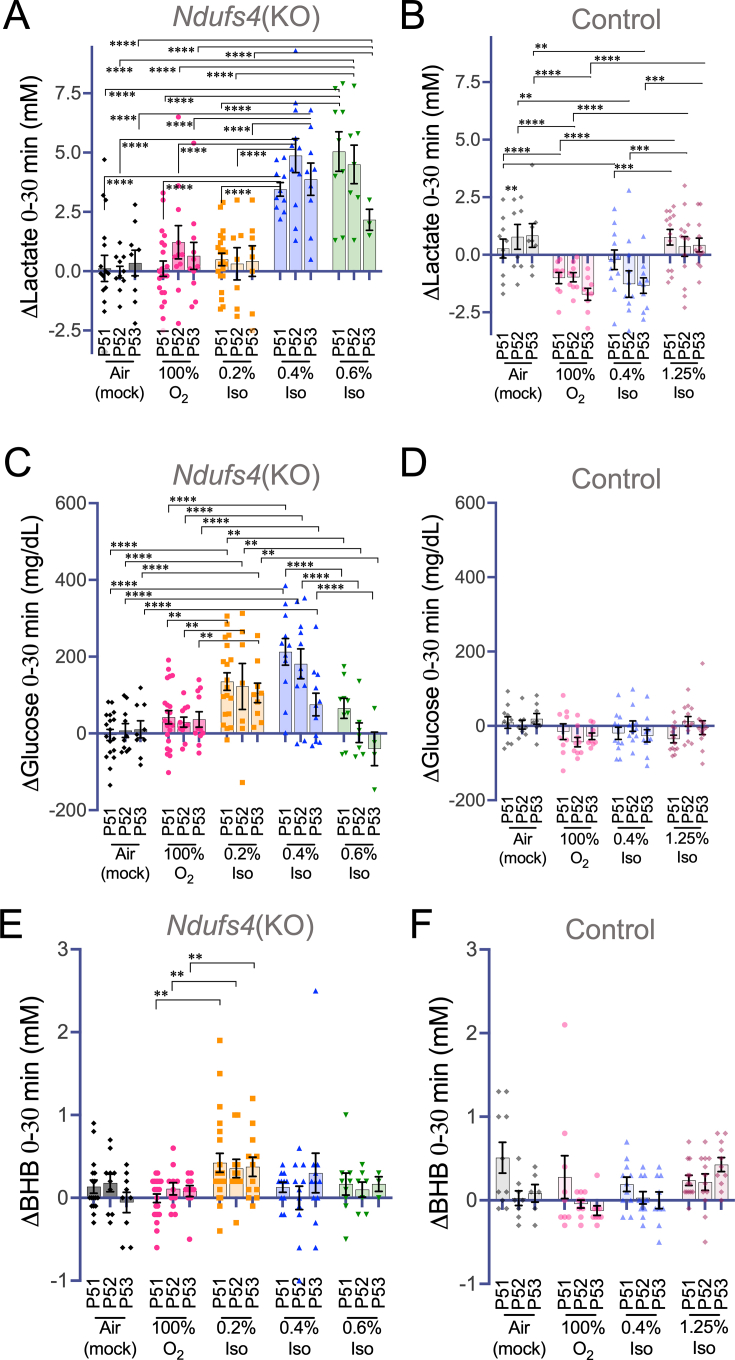


In contrast with righting reflex and ventilatory frequency, there was no significant change in metabolite responses in the second or third exposures in *Ndufs4*(−/−) mice (Supplementary Fig. S3). There were also no changes in responses from one exposure to the next in controls. Comparing all exposures in controls, O_2_ 100% and isoflurane 0.4% both led to slight but significant decreases in blood lactate compared with air or isoflurane 1.25%, whereas no treatment increased lactate (Supplementary Fig. S3).

**Figure S4:**
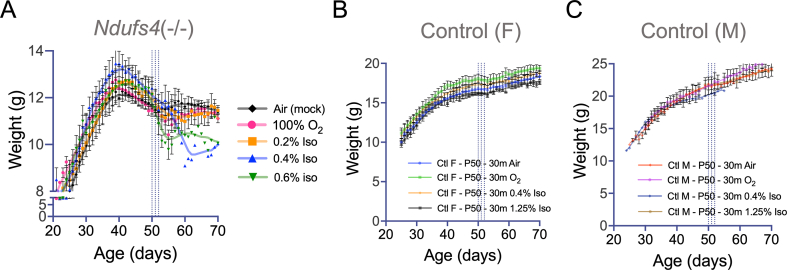


No significant changes in weight were induced by equipotent (1.25%) or equimolar (0.4%) concentrations of isoflurane in control animals (Fig. 2d, Supplementary Fig. S4). In *Ndufs4*(−/−) mice, all concentrations of isoflurane caused significant weight loss (Fig. 2e and f). Loss was significant in all isoflurane exposures compared with air, and in O_2_, 0.2% and 0.6% were significant compared with 100% (0.4% *vs* O_2_ 100%, *P*=0.14).

As noted above, acute mortality was observed after even single exposures to isoflurane 0.6%. Acute mortality was also observed at 0.4% when animals were exposed on consecutive days, and isoflurane accelerated mortality in *Ndufs4*−/−) mice in a concentration-dependent manner; isoflurane 0.4% and 0.6% significantly reduced survival compared with both O_2_ 100% and mock treatment (Fig. 2g and h). In these groups, approximately one-quarter and two-thirds, respectively, died during the P50–52 treatment period. Critically, O_2_ 100% alone did not impact survival. No mortality was observed in any control animal cohort.

### Toxicity is dependent on exposure duration

**Fig 3:**
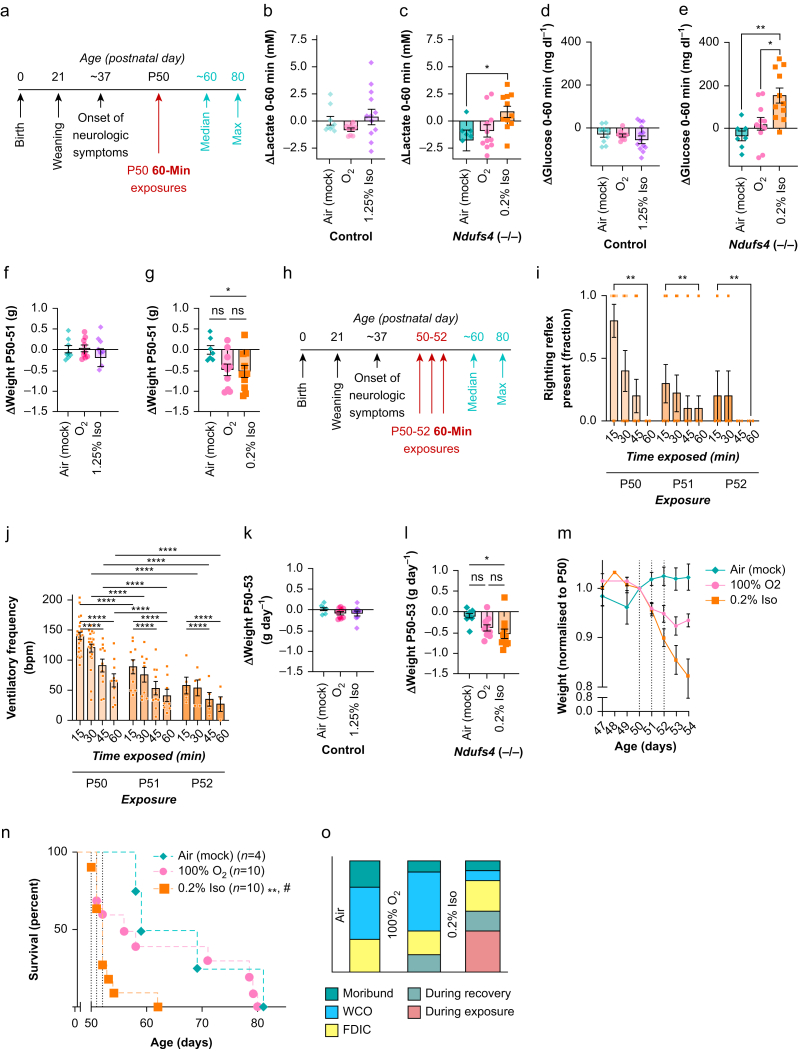
Impact of 60-min isoflurane exposures from P50–P52 in the *Ndufs4*(−/−). (a) Overview of *Ndufs4*(−/−) disease course and the paradigm for 60-min exposure to isoflurane (Iso). (b) Change in blood lactate in control mice during a 60-min exposure at P50 to Iso 1.25%, O_2_ 100%, or air. One-way ANOVA = not significant. (c) Change in blood lactate in *Ndufs4*(−/−) mice during a 60-minute exposure at P50 to Iso 0.2%, O_2_ 100%, or air. One-way analysis of variance (anova) ∗*P*<0.05. (d) Change in blood glucose in control mice during a 60-min exposure at P50 to Iso 1.25%, O_2_ 100%, or air. One-way anova=not significant. (e) Change in blood glucose in *Ndufs4*(−/−) mice during a 60-min exposure at P50 to Iso 0.2%, O_2_ 100%, or air. One-way anova ∗∗*P*<0.005. (b–e) *n*≥7 per group; in (e), one datapoint for the air cohort is outside the plotted range (−7.1 mM; range kept consistent between plots for visual comparison). (f) Change in control animal weight over the 24 h after a single 60-min exposure to Iso 1.25%, O_2_ 100%, or air. One-way anova=not significant. (g) Change in *Ndufs4*(−/−) weight over the 24 h after a single 60-min exposure to Iso 0.2%, O_2_ 100%, or air. One-way anova ∗*P*<0.05. (f–g) *n*≥7 per group. (b–g) *P*-values shown are Tukey's multiple testing corrected p-values: ∗∗∗∗*P*<0.0001, ∗∗∗*P*<0.0005, ∗∗*P*<0.005, ∗*P*<0.05. All datapoints shown. (h) Overview of *Ndufs4*(−/−) disease course and the paradigm for once daily 60-min exposure to Iso at P50, P51, and P52. (i) Loss of righting reflex by exposure day and time exposed in Iso 0.2% in *Ndufs4*(−/−) animals. ∗∗*P*<0.005 (exposure number factor) and ∗∗∗∗*P*<0.00005 (time factor) by Mann–Whitney test. (j) Ventilatory frequency by time into exposure and exposure number in Iso 0.2%-exposed *Ndufs4*(−/−) animals. Two-way anova: time factor ∗∗∗*P*<0.0005, exposure number factor ∗∗∗∗*P*<0.0001. (i–j) *n*≥5 per group except 60-min ventilatory frequency on P53, where *n*=4. *P*-values shown are Tukey's multiple comparisons test ∗*P*<0.05, ∗∗*P*<0.005, ∗∗∗*P*<0.0005, ∗∗∗∗*P*<0.0001. *P*-values for irrelevant comparisons (different duration and day) not shown. (k–l) The rate of change in weight (grams per day, g day^−1^) in control (k) and *Ndufs4*(−/−) mice during the P50–P53 period, calculated using a slope equation with available datapoints; where animals did not survive to P53 the slope was calculated using available days. (k) One-way anova—not significant. (l) One-way anova ∗*P*<0.05. For comparisons shown, ∗*P*<0.05 by Tukey's multiple comparisons test pairwise *P*-value. (k–l) *n*≥6 per group. (m) *Ndufs4*(−/−) weights normalised (by individual animals) to P50, focused on the days spanning the exposures. Plotted are mean with standard error of the mean (sem). No weight changes occurred in controls (see Supplementary Fig. S4). Weights were not compared on individual days, slopes were compared (k–l). (n) Survival of *Ndufs4*(−/−) animals exposed to 60 min of Iso 0.2%, O_2_ 100%, or air once daily on P50, P51, and P52. ∗∗*P*<0.005 against air, ^#^*P*<0.05 against O_2_ 100%, Gehan–Breslow–Wilcoxon test. *n* as listed in legend. (o) Cause of death in *Ndufs4*(−/−) mice from these experiments (those animals in survival curves in [*n*]). (b–m) Error bars=standard error of the mean (sem) centred on the mean. All datapoints represent biological replicates (individual animals). Any pairwise comparisons not shown are non-significant (*P*>0.05). FDIC, found dead in cage; ns, nonsignificant; WCO, euthanised as a result of reaching weight cut-off.

**Figure S5:**
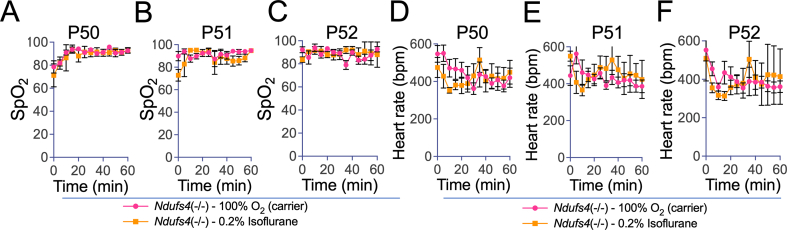


Studies probing sequelae of VA exposure in mice typically expose animals for 2–4 h of deep anaesthesia.^2^^,^^25^^,^^27^ In comparison, the above exposures were brief and moderate. As relevant paediatric surgeries can be quite long,^28^ we next sought to assess the impact of exposure duration. To do so, we used the approaches outlined above but increased exposures to 60–min (Fig. 3a). Given the robust impact of isoflurane 0.2% (details following), only this concentration was tested in *Ndufs4*(−/−) mice in this paradigm. As in 30-min treatments, peripheral blood oxygenation and heart rate were maintained during exposures (Supplementary Fig. S5).

**Figure S6:**
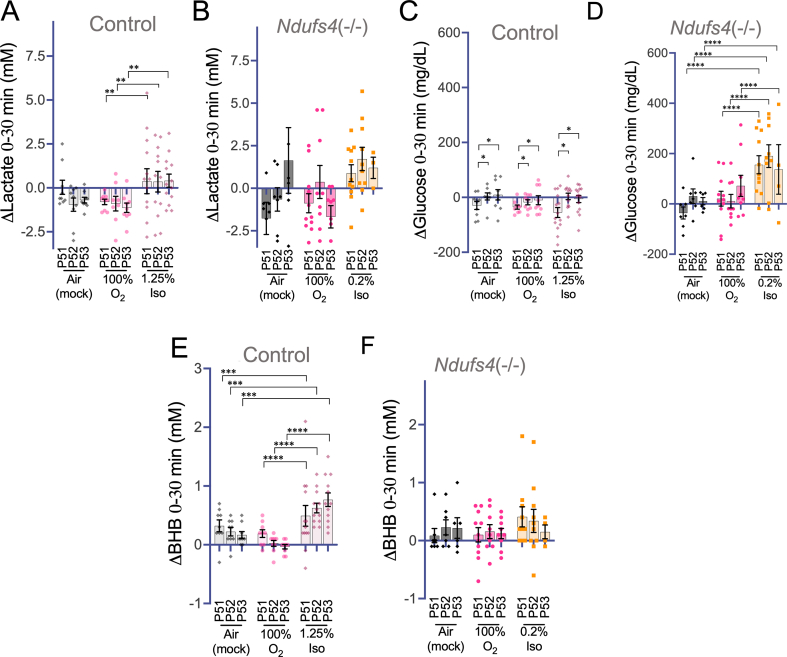


Blood lactate and glucose were both increased by one 60-min exposure to isoflurane 0.2% in *Ndufs4*(−/−) mice compared with air, but not by isoflurane 1.25% in controls (Fig. 3b–d). βHB was mildly increased by isoflurane 1.25% in controls, but not by isoflurane 0.2% in *Ndufs4*(−/−) mice (Supplementary Fig. S6). A 60-min exposure did not impact control animal weight, but a single isoflurane 0.2% exposure led to significant weight loss at 24 h after the exposure in *Ndufs4*(−/−) mice compared with a mock treatment (Fig. 3f and g). Qualitatively, O_2_ exposure also appears to impact weight, but the change was not significant.

**Figure S7:**
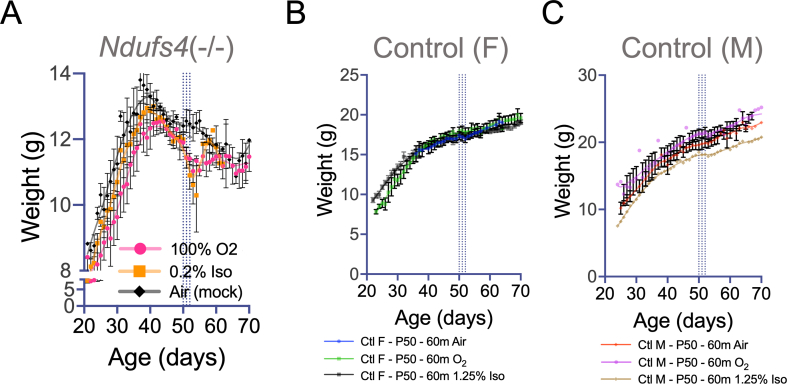


Consistent with the 30-min paradigm, *Ndufs4*(−/−) sensitivity to LORR and to ventilatory frequency depression was increased on second and third exposures (Fig. 3i and j). Daily 60-min exposures to isoflurane did not impact weight in control mice, but isoflurane 0.2% led to significant weight loss in *Ndufs4*(−/−) mice during the P50–53 period compared with the mock treatment cohort (Fig. 3k–m, Supplementary Fig. S7). Intermediate, non-significant, weight loss occurred in O_2_ 100%-exposed *Ndufs4*(−/−) mice.

The 60-min exposures to isoflurane 0.2% significantly shortened survival compared with either O_2_ 100% or air (mock) treatments, with >80% mortality during the exposure period (Fig. 3n and o). The 60-min exposures to O_2_ 100% also resulted in acute mortality: 30% before the second exposure, and an additional 10% after the second, indicating that O_2_ 100% is not benign in ETC CI MD (see below, Discussion). However, overall survival in this group was not reduced compared with the mock (air) cohort, and mortality did not appear accelerated after the treatment period.

### Isoflurane toxicity is limited in Ndufs4(−/−) mice at ages preceding CNS disease onset

**Fig 4:**
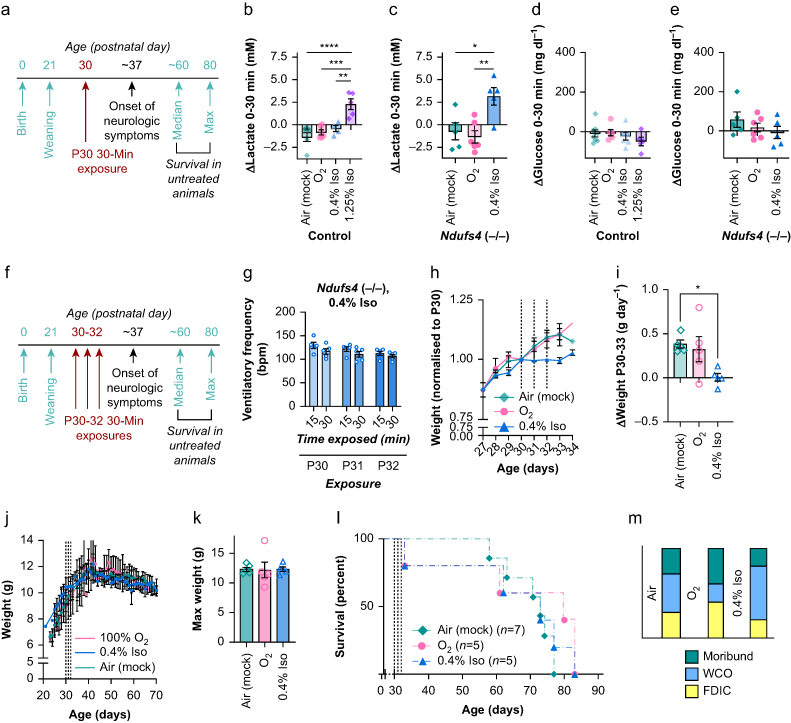
Isoflurane (Iso) toxicity in the *Ndufs4*(−/−) is limited at ages preceding onset of neurologic disease. (a) Overview of the onset of disease in the *Ndufs4*(−/−) and the relative timing of P30 30-min exposures. (b) Change in blood lactate in control mice during a 30-min exposure at P30 to Iso 0.4% or 1.25%, O_2_ 100%, or air. One-way analysis of variance (anova) ∗∗∗*P*<0.0001. (c) Change in blood lactate in *Ndufs4*(−/−) mice during a 30-min exposure at P30 to Iso 0.4%, O_2_ 100%, or air. One-way anova ∗∗*P*<0.005. (d) Change in blood glucose in control mice during a 30-min exposure at P30 to Iso 0.4% or 1.25%, O_2_ 100%, or air. One-way anova=not significant. (e) Change in blood glucose in *Ndufs4*(−/−) mice during a 30-min exposure at P30 to Iso 0.4%, O_2_ 100%, or air. One-way anova=not significant. (b–e) *n*≥5 per group. (f) Overview of the onset of disease in the *Ndufs4*(−/−) and the relative timing of repeat (once daily) P30, P31, and P32 30-min exposures. (g) Ventilatory frequency at 15 and 30 min of exposure by exposure number in Iso 0.4%-exposed cohort. Data compared by two-way anova: time factor and exposure number factor were both non-significant. No Tukey's multiple comparisons test pairwise comparisons (all possible combinations tested) reached significance (Tukey adjusted P<0.05). See Figure 1c for comparison with Iso 0.2% at P50. (h) *Ndufs4*(−/−) weights normalised (by individual animals) to P30, focused on the days spanning the exposures. Plotted are mean with standard error of the mean (sem). Weights were not compared on individual days, slopes were compared in (i). We observed no weight changes in controls. (i) The rate of change in weight (grams per day, g day^−1^) in *Ndufs4*(−/−) mice during the P30–P33 period, calculated using a slope equation. One-way anova ∗*P*<0.05. Pairwise comparison ∗*P*<0.05 by Tukey's multiple comparisons test. (j) Overall weight plots for *Ndufs4*(−/−) mice exposed to Iso, O_2_ 100%, or air on P30, P31, and P32. Weight changes were transient, with no overall impact on weight gain or subsequent loss during normal disease progression starting around P37. Plotted are mean and standard error of the mean for each day, lines are LOWESS (locally weighted scatterplot smoothing) running averages. (k) Maximum weight of *Ndufs4*(−/−) mice was not impacted by exposure to Iso or O_2_ 100%, compared with air, on P30, P31, and P32. One-way anova—not significant. No pairwise comparisons significant by Tukey's multiple comparisons test. (l) Survival of *Ndufs4*(−/−) animals exposed to Iso 0.4%, O_2_ 100%, or air 30 min per day from P30 to P32. No significant differences in survival were detected by Gehan–Breslow–Wilcoxon test, and survival curves overlap. (m) Cause of death in *Ndufs4*(−/−) mice from these experiments (those animals in survival curves in (l). (g–m) *n* on each *Ndufs4*(−/−) dataset are reflected in the survival curve in (l). FDIC, found dead in cage; WCO, euthanised as a result of reaching weight cut-off.

**Figure S8:**
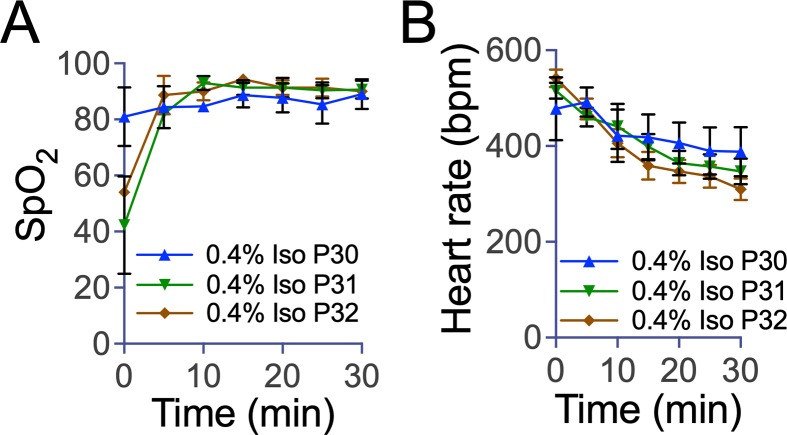


As detailed above, progressive inflammatory brainstem lesions are a defining feature of Leigh syndrome. Disease typically onsets in the first years of life in humans,^7^^,^^29^ and at ∼P37 in *Ndufs4*(−/−) mice. CNS lesions have not been detected before this age.^12^ To assess whether isoflurane toxicity is contingent on the presence of overt neurodegenerative disease, we exposed pre-disease onset *Ndufs4*(−/−) mice at age P30 to 30 min per day isoflurane 0.4%, O_2_ 100%, or air (Fig. 4a). As in older animals, heart rate and SpO_2_ were unremarkable (Supplementary Fig. S8).

**Figure S9:**
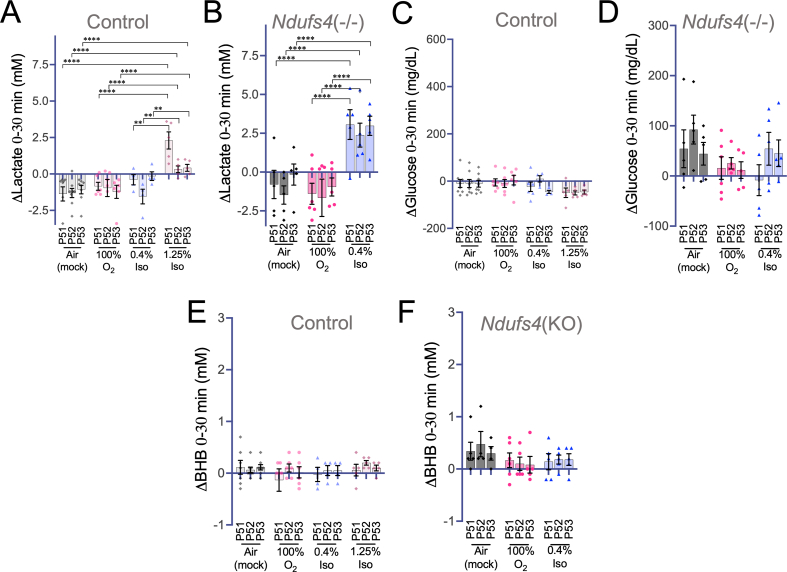


Blood lactate was significantly increased by exposure to isoflurane 0.4% in *Ndufs4*(−/−) mice at P30, as seen at P50 (Fig. 4b and c). Interestingly, lactate was similarly increased in controls exposed to isoflurane 1.25%. In contrast to P50, glucose was not changed in control or *Ndufs4*(−/−) mice (Fig. 4d and e). βHB was unchanged in all groups, and all metabolite changes were similar in repeat exposures (Supplementary Fig. S9).

At P50, 0.2% caused significant respiratory depression in *Ndufs4*(−/−) mice, with increasing effect in repeat exposures. In contrast, ventilatory frequency was not significantly depressed or impacted by exposure number during repeat exposures to the higher concentration of 0.4% from P30–32 (Fig. 4g).

At P30, mice are gaining weight as part of normal postnatal growth. The 30-min exposures to isoflurane 0.4% on P30, P31, and P32 reduced weight gain during this period but had no lasting effect on maximum weight or overall weight curves (Fig. 4i–k). Furthermore, exposures to isoflurane 0.4% at pre-disease onset ages of P30–P32 did not significantly alter survival of *Ndufs4*(−/−) mice (Fig. 4l and m), though a low rate of acute mortality cannot be ruled out.

### Anaesthesia toxicity is largely prevented by colony stimulating factor 1 receptor inhibition

**Fig 5:**
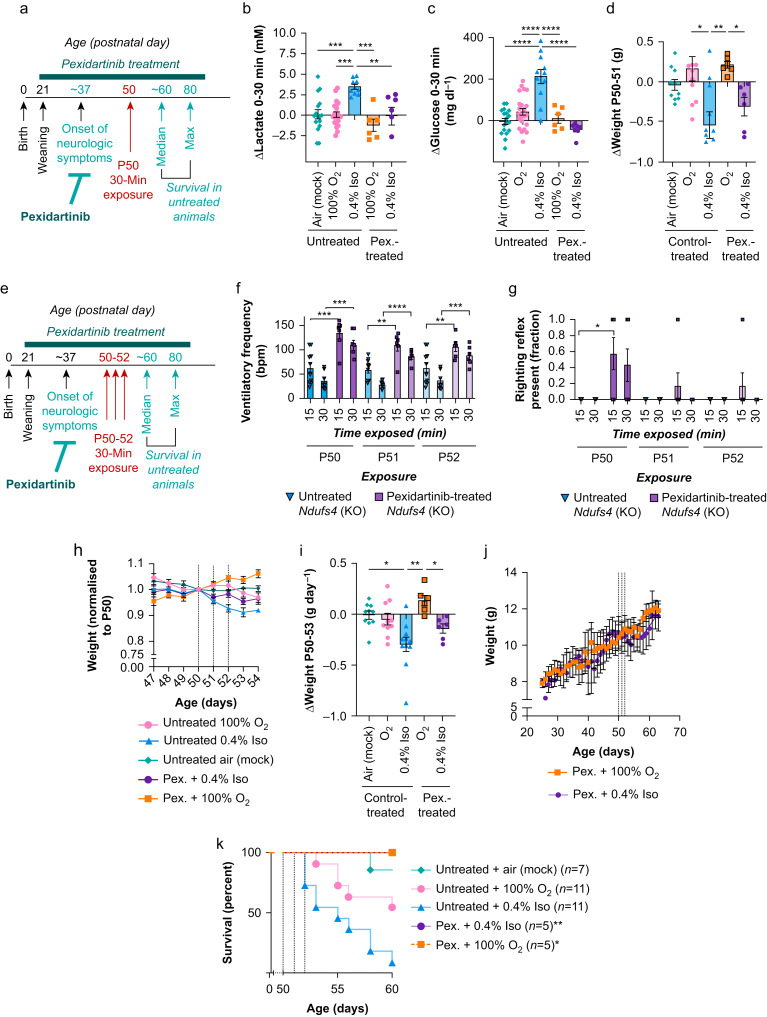
Pexidartinib treatment prevents or attenuates toxic effects of isoflurane (Iso) in the *Ndufs4*(−/−). (a) Overview of the onset of disease in the *Ndufs4*(−/−) and the relative timing of pexidartinib treatment and P50 exposures. (b) Changes in blood lactate in pexidartinib treated and control diet-treated *Ndufs4*(−/−) mice during a 30-min exposure at P50 to Iso 0.4%, O_2_ 100%, or air. One-way analysis of variance (anova) ∗∗∗∗*P*<0.0001. Tukey's multiple testing corrected pairwise comparison *P*-values ∗∗*P*<0.005, ∗∗∗*P*<0.0005. (c) Changes in blood glucose in pexidartinib-treated and control diet-treated *Ndufs4*(−/−) mice during a 30-min exposure at P50 to Iso 0.4%, O_2_ 100%, or air. One-way anova ∗∗∗∗*P*<0.0001. Tukey's multiple testing corrected pairwise comparison *P*-values ∗∗∗∗*P*<0.0001. (d) Change in pexidartinib-treated and control diet-treated *Ndufs4*(−/−) weight over the 24 h after a single 30-min exposure at P50 to Iso 0.4%, O_2_ 100%, or air. One-way anova ∗∗∗*P*<0.0005. Tukey's multiple testing corrected pairwise comparison *P*-values ∗*P*<0.05, ∗∗*P*<0.005. (b–d) *n*≥6 per group. (e) Overview of the onset of disease in the *Ndufs4*(−/−) and the relative timing of pexidartinib treatment and daily Iso 0.4%, O_2_ 100%, or air, exposures on P50, P51, and P52. (f) Ventilatory frequency at 15 and 30 min of exposure to Iso 0.4% in pexidartinib-treated or control diet-treated *Ndufs4*(−/−) mice. Two-way anova: exposure number factor ∗∗∗∗*P*<0.0001, treatment factor ∗∗∗∗*P*<0.0001. Pairwise comparisons by Tukey's multiple comparison test: ∗∗*P*<0.005, ∗∗∗*P*<0.0005, ∗∗∗∗*P*<0.00005. (g) Righting reflex presence in Iso 0.4% exposures in pexidartinib-treated or control diet-treated *Ndufs4*(−/−) mice. 1=righting reflex present (animals un-anaesthetised), 0=righting reflex absent (animals are anaesthetised to the point of loss of righting reflex). ∗*P*<0.05 by paired non-parametric Mann–Whitney test. (h) Weights of pexidartinib-treated and control diet-treated *Ndufs4*(−/−) animals exposed to Iso 0.4%, O_2_ 100%, or air on P50, P51, and P52, normalised to P50. Data are mean with standard error of the mean (sem). (i) The rate of change in weight (grams per day, g day^−1^) during the P50–P53 period. Datapoints represent individual animals, with bars at the mean and error bars representing sem. One-way anova ∗∗∗*P*<0.001 by. Tukey's multiple testing corrected pairwise comparison *P*-values ∗*P*<0.05, ∗∗*P*<0.005, ∗∗∗*P*<0.0001. (j) Raw weight plots of pexidartinib-treated *Ndufs4*(−/−) mice exposed to O_2_ 100% or Iso 0.4% (in O_2_ 100%) throughout the P50–P52 exposure period. Data are mean with error bars showing sem, lines are LOWESS (locally weighted scatterplot smoothing) running averages. (k) Survival of untreated and pexidartinib-treated *Ndufs4*(−/−) animals exposed to 30 min of Iso 0.4% or O_2_ 100% at P50–P52. ∗*P*<0.05 and ∗∗*P*=0.002 *vs* untreated *Ndufs4*(−/−) mice exposed to Iso 0.4% or O_2_ 100%, respectively, by Gehan–Breslow–Wilcoxon test, with pexidartinib-treated mouse cohort halted when pexidartinib reached P60–P80. No pexidartinib-treated animals showed signs of disease at study end. (a–k) Pexidartinib group *n* ≥ those indicated in panel (k). Control diet-treated groups as detailed in Fig 1, Fig 2. All datapoints represent individual animals, all error bars represent sem. KO, knockout; Pex., pexidartinib.

Recently, we reported that PLX3397/pexidartinib, a CSF1R (colony stimulating factor 1 receptor) inhibitor which depletes macrophages (including microglia), prevents disease in the *Ndufs4*(−/−), including a full suppression of CNS lesions.^8^ Given that isoflurane-related toxicities are attenuated or absent in *Ndufs4*(−/−) animals before CNS disease onset, we next assessed whether treatment with pexidartinib would alter toxicity in the P50–P52, 30-min paradigm. *Ndufs4*(−/−) animals were treated with pexidartinib 300 mg/kg/day and exposed to isoflurane at isoflurane 0.4% as in Fig 1, Fig 2 (Fig. 5a).

**Figure S10:**
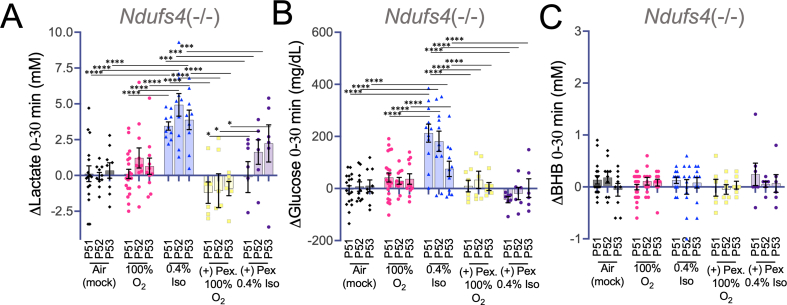


Pexidartinib treatment prevented changes to blood lactate and blood glucose induced by a single exposure to isoflurane 0.4% (Fig. 5b and c, Supplementary Fig. S10). However, weight loss induced by one exposure was only partly (non-significantly) attenuated. On all three exposure days, ventilatory frequency depression was prevented by pexidartinib treatment (Fig. 5f). Anaesthesia sensitivity, by righting reflex, was reduced in pexidartinib-treated animals during the first exposure (P50) (Fig. 5g).

Isoflurane 0.4% led to a modest reduction in the weight of pexidartinib treated *Ndufs4*(−/−) mice compared with pexidartinib-treated oxygen 100%-exposed animals (Fig. 5h–j). The loss was reduced compared with untreated, isoflurane 0.4%-exposed, *Ndufs4*(−/−) mice, and was transient (weight recovered). Critically, all pexidartinib-treated *Ndufs4*(−/−) animals survived the exposure paradigm. These cohorts ended after P60 when nearly all of the untreated/isoflurane 0.4%-exposed, and 50% of the untreated/O_2_ 100%-exposed animals had died. At this age, the pexidartinib-treated animals were free of disease (no signs of weight loss, ataxia, or neurological disease^8^) with no signs of chronic toxicities resulting from the isoflurane exposures (Fig. 5k).

### Impact of oxygen in the carrier gas

O_2_ 100% is the standard anaesthetic carrier gas in laboratory animal research and veterinary medicine, though recent studies suggest lower oxygen concentrations may be beneficial and human medicine has moved to lower concentrations.^30^^,^^31^ Given the weight loss and early mortality we observed in the P50, 60-min O_2_ 100% cohort, we next tested the impact of reduced carrier gas oxygen on isoflurane toxicity in the *Ndufs4*(−/−).

**Fig 6:**
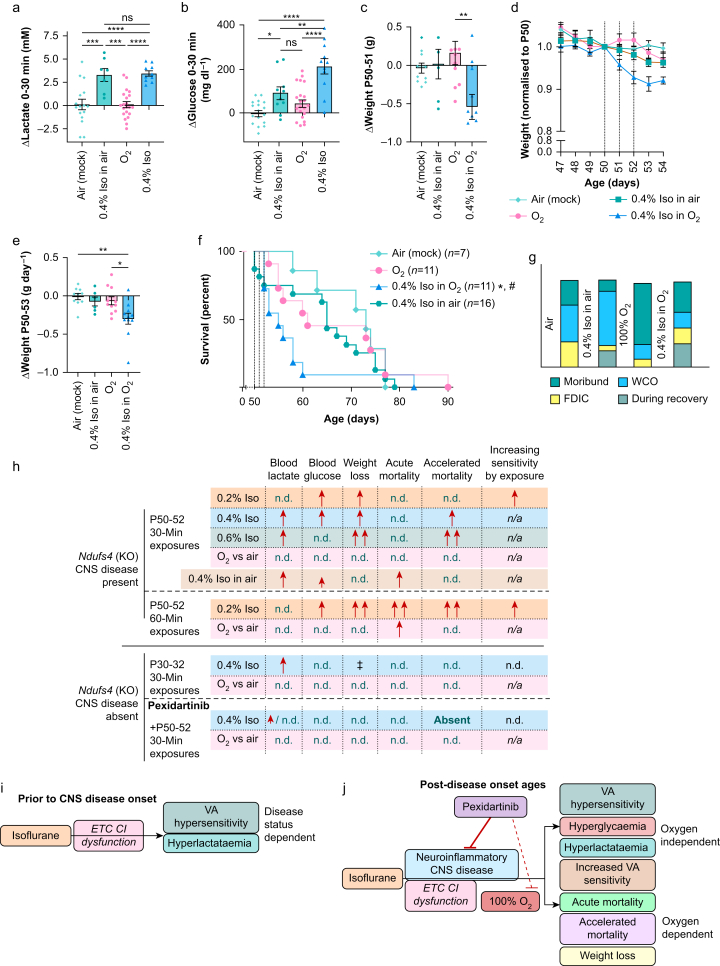
Carrier gas oxygen concentration impacts the toxicity of isoflurane (Iso) 0.4% in *Ndufs4*(−/−) mice. (a) Changes in blood lactate in *Ndufs4*(−/−) mice during a 30-min exposure at P50 to Iso 0.4% in O_2_ 100% or air, and in O_2_ 100% or air alone. One-way analysis of variance (anova) ∗∗∗∗*P*<0.0001. Tukey's multiple testing corrected pairwise comparison *P*-values ∗∗∗*P*<0.0005, ∗∗∗∗*P*<0.0001. (a) Changes in blood glucose in *Ndufs4*(−/−) mice during a 30-min exposure at P50 to Iso 0.4% in O_2_ 100% or air, and in O_2_ 100% or air alone. One-way anova ∗∗∗∗*P*<0.0001. Tukey's multiple testing corrected pairwise comparison *P*-values ∗*P*<0.05, ∗∗*P*<0.005, ∗∗∗∗*P*<0.0001. (c) Change in weight over the 24 h after a single 30-min exposure at P50 to Iso 0.4% in O_2_ 100% or air or O_2_ or air alone. One-way anova ∗*P*<0.05; Tukey's multiple testing corrected pairwise comparison *P*-values ∗∗*P*<0.005. (d) Average weights, normalised to P50 by animal, of *Ndufs4*(−/−) mice exposed Iso 0.4% in O_2_ 100% or air, or O_2_ 100% or air alone, once daily from P50 to P52. Individual days not compared; rates of weight change compared in (e). (e) Rate of weight change from P50 to P53 in *Ndufs4*(−/−) animals exposed to Iso 0.4% in O_2_ 100% or air or O_2_ or air alone. One-way anova ∗∗*P*<0.005; Tukey's multiple testing corrected pairwise comparison *P*-values ∗*P*<0.05, ∗∗*P*<0.005. (f) Survival of *Ndufs4*(−/−) animals exposed to 30 min of Iso 0.4% in O_2_ 100% or air, or O_2_ 100% or air alone, once daily from P50 to P52. ∗*P*<0.05 *vs* air and ^#^*P*<0.05 *vs* O_2_ 100% by Gehan–Breslow–Wilcoxon test. Early mortality in Iso 0.4% in air was significant compared with historic data on unexposed animals during these ages (Supplementary Fig. S12). (g) Cause of death from mice in (f). For all groups and panels, *n* ≥7. Cohorts other than Iso 0.4% in air appear in Fig 1, Fig 2. (h) Summary of findings in this study. Isoflurane exposure results in concentration- and duration-dependent toxicities in the *Ndufs4*(−/−) model of Leigh syndrome including hyperlactataemia, hyperglycaemia, weight loss, and accelerated mortality. O_2_ 100% resulted in acute mortality in 60-min exposures, but in most outcomes the carrier gas alone was benign. Notably, isoflurane 0.2% borders on providing anaesthesia in the first exposure (Fig. 1), but increased sensitivity to anaesthesia was observed in repeat exposures. Few toxic sequelae were present in Iso 0.4%-exposed *Ndufs4*(−/−) mice when exposed at pre-CNS disease onset ages, or in animals treated with the macrophage/microglia-depleting drug pexidartinib. Absent—mortality absent for the duration of the experiment, survival is extended compared with untreated *Ndufs4*(−/−). (i–j) Diagrammatic models of isoflurane toxicities at pre-CNS disease onset ages (i) and post-disease onset ages (j). (i) At all ages, *Ndufs4*(−/−) mice show hypersensitivity to anaesthesia and elevation of lactate. (j) After disease onset, additional toxic sequelae arise: hyperglycaemia, weight loss, accelerated mortality, and increased sensitivity in subsequent exposures. Pexidartinib is known to prevent CNS lesions in this model, and prevents Iso toxicity. However, hyperlactataemia and volatile anaesthetic hypersensitivity occur even in the absence of overt disease but are attenuated by pexidartinib, suggesting a role for immune cells even in the absence of overt CNS degeneration. ^‡^Weight gain was prevented during the exposure period, but no weight loss occurred. ETC CI, electron transport chain complex I; FDIC, found dead in cage; KO, knockout; n.d., not detected (no statistically significant effect); n/a, not applicable (i.e. these animals were either anaesthetised even on the first exposure, or were oxygen treated so never anaesthetised); ns, nonsignificant; VA, volatile anaesthetic; WCO, euthanised as a result of reaching weight cut-off.

**Figure S11:**
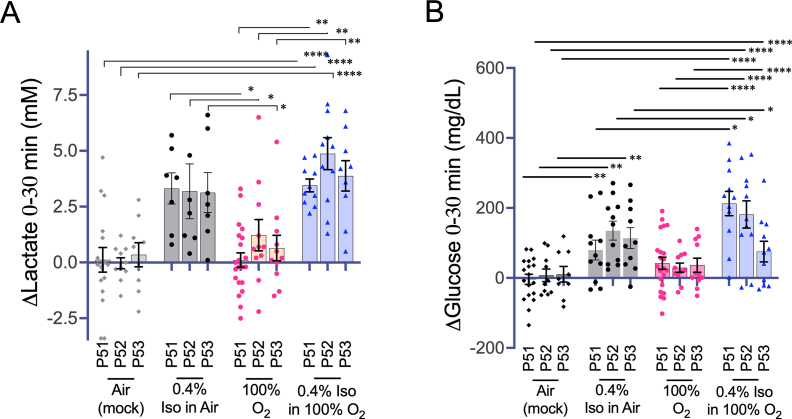


**Figure S12:**
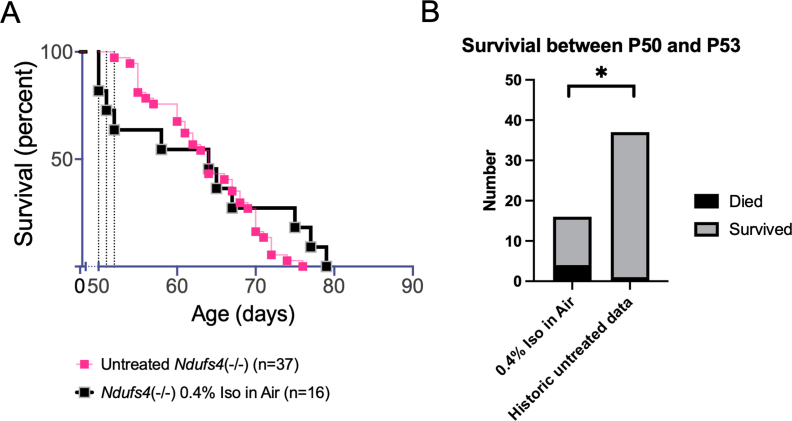


The increase in blood lactate induced by isoflurane 0.4% was not altered by using medical air (O_2_ 21%), rather than O_2_ 100%, as a carrier gas, while isoflurane induced hyperglycaemia was partly attenuated (Fig. 6a and b, Supplementary Fig. S11). Weight loss induced by one or multiple exposures to isoflurane 0.4% was rescued by the use of medical air (Fig. 6c–e). Significant acute mortality was observed in the isoflurane 0.4% in medical air group: 36% of this cohort died during the 3-day exposure paradigm, higher than air or O_2_ 100% only (Fig. 6f and g), and significantly increased compared with untreated (no exposure) *Ndufs4*(−/−) animals (Supplementary Fig. S12). However, overall survival of this cohort was not significantly reduced compared with the air or O_2_ 100% groups, in contrast with isoflurane 0.4% in air (Fig 1, Fig 2), with no apparent acceleration of mortality among animals that survived the treatment period.

## Discussion

### Toxicities of anaesthesia in the Ndufs4(−/−)

Here, we have assessed the impact of isoflurane in the *Ndufs4*(−/−) model of Leigh syndrome, summarised in Figure 6h–j. Our data reveal that when neurological symptoms are present, exposure to isoflurane results in concentration- and duration-dependent toxicities including acute hyperlactataemia and hyperglycaemia, acute weight loss, and increased sensitivity to subsequent exposures, determined by LORR and ventilatory frequency. Survival is reduced in a concentration- and duration-dependent manner. Importantly, toxicities were present even at 0.2%, which was insufficient for complete anaesthesia by righting reflex.

In contrast, exposure to an anaesthetising concentration of isoflurane at P30, pre-disease onset, was generally benign. Given that mitochondrial function (ETC CI enzymatic activity) does not change with age in this model,^32^ these data indicate that the presence of neuroinflammatory pathology in post-disease onset animals contributes significantly to anaesthetic toxicities (see Stokes and colleagues^8^ and Supplementary Fig. S13 for images of brainstem lesions). Consistent with this interpretation, treatment with the CSF1R inhibitor pexidartinib, which prevents lesion formation and disease in the *Ndufs4*(−/−), broadly rescued anaesthesia toxicity at P50.

Interestingly, isoflurane-induced hyperlactataemia occurred at both pre- and post-disease onset ages, but pexidartinib prevented the isoflurane-induced lactate increase. These data indicate that this effect of isoflurane is independent of disease status/age in the *Ndufs4*(−/−) but mediated by pexidartinib-targeted cells.

Our data support the following conclusions: first, VA exposure is not benign in the setting of ETC CI dysfunction when overt disease is present. Second, toxicity is strongly influenced by the presence of neurologic disease, as toxicities are significantly reduced at pre-disease ages and in pexidartinib-treated animals. Third, isoflurane toxicity in ETC CI dysfunction is largely mediated by actions of immune cells targeted by pexidartinib.

Interestingly, the benefits of pexidartinib on blood lactate and anaesthesia sensitivity appear pathology-independent, as these are influenced by pexidartinib treatment but not disease status. Conversely, it is possible that pexidartinib rescues underlying subclinical immune dysfunction present before the onset of overt disease. Future studies using genetic approaches to eliminate immune cells or conditionally ablate *Ndufs4* may help define the relative importance of neuroinflammatory CNS disease in overt LS *vs* other roles for pexidartinib-targeted cells.

### Contribution of oxygen

In human clinical anaesthesia, lowering carrier gas O_2_ from 100% has been found to benefit postoperative outcomes, though opinions on the ideal concentration are mixed.^33^, ^34^, ^35^ In contrast, in animal research and veterinary medicine O_2_ 100% is considered standard of care.^30^^,^^31^ The impact of inhaled O_2_ concentration on anaesthetic outcomes in animals has not been rigorously explored, though some data,^30^^,^^36^ including our own,^1^ suggest O_2_ 100% is not overtly detrimental in normal animals.

Here, we find isoflurane is toxic in the *Ndufs4*(−/−) in a concentration-dependent manner with O_2_ 100% used for all concentrations, demonstrating isoflurane itself is toxic (Fig. 6h). However, weight loss and early mortality occurred in longer (60-min) O_2_ 100% only exposures, showing O_2_ is not benign in mitochondrial disease. This is consistent with reports that extended (days) exposures to hyperoxia are toxic to *Ndufs4*(−/−) animals.^10^ Here, we find that medical air (O_2_ concentration of 21%) significantly reduced weight loss and improved long-term survival. In contrast, acute hyperlactataemia was not rescued, hyperglycaemia was only partly attenuated, and acute mortality appeared increased when air was used as the anaesthetic carrier. Accordingly, O_2_ 100% seems to have both positive and negative effects on anaesthetic outcomes compared with O_2_ 21%, though overall it appears that the ideal O_2_ concentration is <100%. Taken more broadly, our findings support other recent studies suggesting a re-evaluation of the use of O_2_ 100% for laboratory animal and veterinary anaesthesia.

### Unanswered questions

Our findings here provide a whole-animal examination of VA toxicity in ETC CI disease. Cellular and tissue changes induced by anaesthesia exposure, and identification of molecular mechanistic underpinnings of anaesthetic targets, will require extensive further study. The variance in lesion size and limited methodology for quantifying lesion volume precludes quantification of anaesthesia-induced changes in lesions. Sensitive *in vivo* methods might allow for more longitudinal experiments, but current techniques, such as MRI, lack the necessary resolution and are limited by their requirement for anaesthesia. Similarly, cognitive function may be impacted by VA exposure, but *Ndufs4*(−/−) animals at P50 suffer from such limited mobility that cognitive function could not be ascertained from standard learning and memory assays. Future studies aimed at assessing the impact on cognitive outcomes will require sensitive methods, careful interpretation, and perhaps less severe models of disease.

While this is a direct, well controlled, study of VA toxicity in mitochondrial disease, a 2016 review of 111 clinical reports identified a variety of anaesthesia complications in mitochondrial disease patients.^16^ Among these are increased blood lactate and worsening of respiratory dysfunction, both observed here. Our findings are specific to ETC CI dysfunction. Whether genetically distinct forms of disease show similar toxicities remains to be determined. A detailed re-analysis of available clinic data with a focus on genetic causes may provide some answers, but further experimental research will also be necessary.

Recent work has demonstrated that VAs impair excitatory neurotransmission, in particular synaptic vesicle endocytosis, and that this contributes to anaesthesia hypersensitivity in the *Ndufs4*(−/−).^37^^,^^38^ Whether direct effects of VAs on synaptic transmission play any immediate role in anaesthesia toxicities we report here is not yet known. Similarly, the role of reduced NADH (nicotinamide adenine dinucleotide) redox, which has been shown to underlie a portion of disease in the setting of ETC CI deficiency,^39^ remains to be defined in the context of anaesthesia toxicity. Given the benefits of pexidartinib and dependence on disease status, we suspect that these cell and molecular processes lie upstream of immune activity, which proximally drives the majority of toxicity. Future studies will be needed to directly test this model.

## Authors’ contributions

Contributed to project conception, design, acquisition of data, interpretation of data, or all: all authors.

Contributed to manuscript drafting, approve the version for publication, and are accountable for the integrity of the published work: all authors.

Obtained funding: SCJ, MS, PGM, KAS.

Conceived and designed the experiments: SCJ.

Interpreted data, managed the work, and prepared the manuscript: SCJ.

Contributed to manuscript preparation: MS, PGM, KAS, MM, JS, MH, KJ, ARH.

## Acknowledgements

We dedicate this study to Ivy Ohlgren, Colleen and Derek Ohlgren, their family, and all those living with the consequences of mitochondrial disease. They continue to inspire us.

## Declaration of interest

The authors declare that they have no conflicts of interest.

## Funding

US National Institutes of Health (NIH/GM R01 GM133865 to MS, SCJ; NIH/GM R01 GM144368 to SCJ; T32 GM086270 to KAS; R35 GM139566 to PGM); Northwest Mitochondrial Research Guild (to SCJ).

## Figures and Tables

**Figure:**
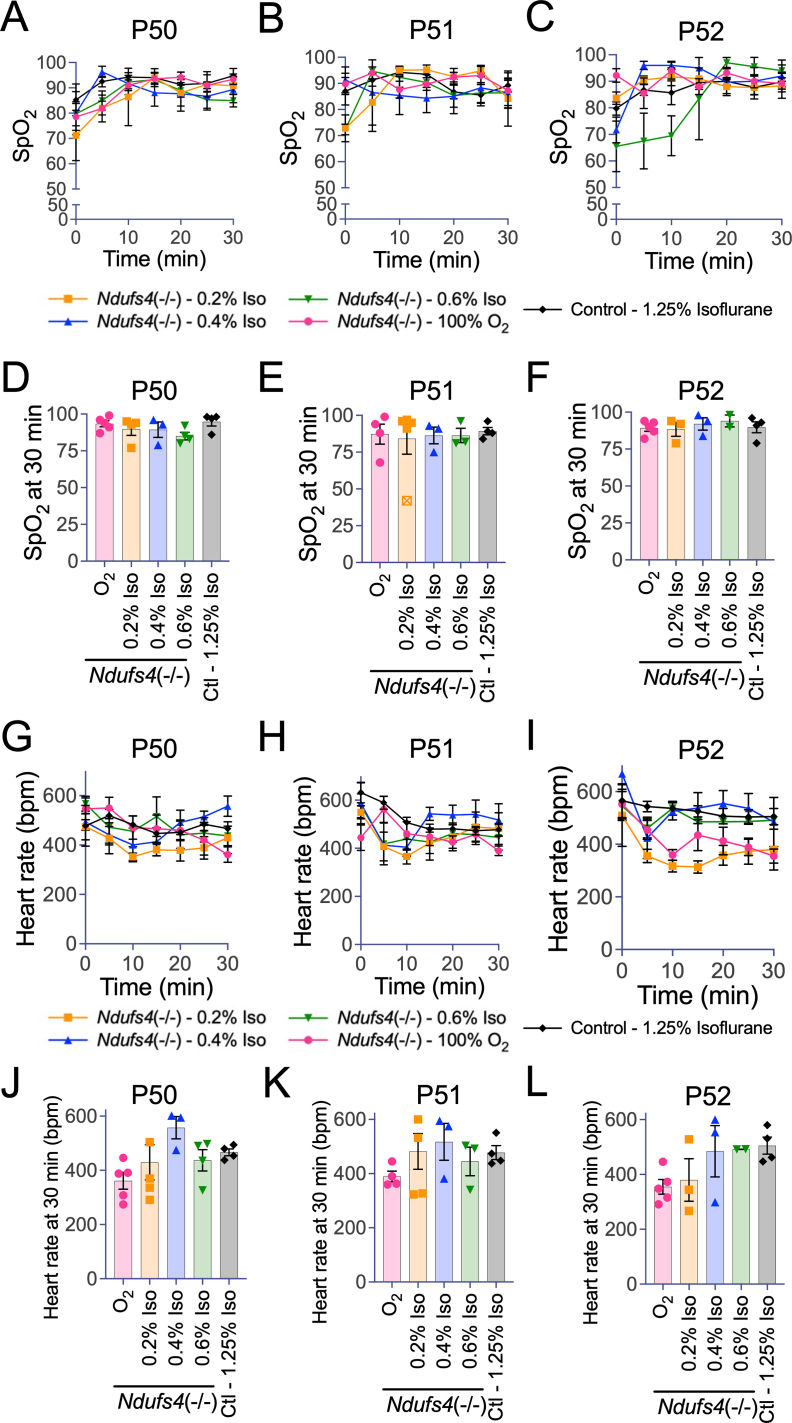
Figure S1

**Figure S13:**